# The association and predictive value analysis of metabolic syndrome combined with resting heart rate on cardiovascular autonomic neuropathy in the general Chinese population

**DOI:** 10.1186/1758-5996-5-73

**Published:** 2013-11-17

**Authors:** Yu Lu, Zi-Hui Tang, Fangfang Zeng, Yiming Li, Linuo Zhou

**Affiliations:** 1 Department of Endocrinology and Metabolism, Fudan University Huashan Hospital, Shanghai 200040, China; 2 Department of Emergency, The 1st affiliated hospital of South China University, Hengyang, Hunan 421001, China

**Keywords:** Metabolic syndrome, Resting heart rate, Cardiovascular autonomic neuropathy, Association, Predictive value

## Abstract

**Background:**

The purpose of this study was to explore the extent of associations of cardiovascular autonomic neuropathy (CAN) with metabolic syndrome (MetS) and resting heart reate (HR), and to evaluate the predictive value of MetS combined with HR on CAN in a large sample derived from a Chinese population.

**Materials and methods:**

We conducted a large-scale, population-based, cross-sectional study to explore the relationships of CAN with MetS and resting HR. This study included 2092 participants aged 30–80 years, and a total of 387 subjects were diagnosed with CAN in our dataset. The associations of CAN with MetS and resting HR were assessed by a multivariate logistic regression (MLR) analysis (using subjects without CAN as a reference group) after controlling for potential confounding factors. The predictive performance of resting HR and MetS was evaluated using the area under the receiver-operating characteristic curve (AUC).

**Results:**

A tendency toward increased CAN prevalence with increasing resting HR was reported (p for trend < 0.001). MLR analysis showed that MetS and resting HR were very significantly and independently associated with CAN (β = 0.495 for MetS and β = 0.952 for HR, P < 0.001 for both). Resting HR alone and combined with MetS (MetS-HR) strongly predicted CAN (AUC = 0.719, P < 0.001 for resting HR and AUC = 0.735, P < 0.001 for MetS-HR).

**Conclusion:**

Our findings signify that MetS and resting HR were very significantly and independently associated with CAN in the general Chinese population. Resting HR and MetS-HR both have a high value in predicting CAN in the general population.

## Background

The prevalence of cardiovascular autonomic neuropathy (CAN) is rapidly growing in all populations worldwide, particularly in the developing world [1,2]. The disease is not only a major factor in the cardiovascular complications of diabetes mellitus (DM) [3], but also affects many other majority segments of the general population, such as the elderly, patients with hypertension (HT), Metabolic syndrome (MetS) and connective tissue disorders [1,4-6]. CAN has become a major health concern in China following recent, rapid changes in lifestyles. In diabetic patients, the prevalence of CAN was 30-60% [3]. Individuals with previously undiagnosed CA dysfunction have an unfavorable cardiovascular risk profile, especially in terms of sudden death, indicating a higher risk of cardiovascular disease [7,8]. MetS is also a global health concern and refers to a constellation of the risk factors of cardiovascular disease, including obesity, abdominal fat distribution, disorders of glucose and lipid metabolism and HT [9]. The burden of MetS is likely to continue to rise, largely due to a decrease in physical activity and an increase in obesity in our society [10].

Previous studies indicate that increased resting HR is an early indicator of CAN, and a strong association between HR and CAN was found in diabetic patients [11,12]. Moreover, in diabetic patients, resting HR is considered a critical clinical early biomarker for CAN. In diabetic patients, MetS was also reported to be associated with CA function indices or impaired CA function [4,13,14]. These studies involved in the association of MetS and HR with CAN were conducted in subgroup level such as diabetic patients. In our previous study, in a Chinese population, MetS was found to be associated with components of CA function [15]. If we can clarify the relationship of CAN with MetS and resting HR in the general population, this information may help clinicians in the prediction, prevention and treatment of CAN.

However, at the population level, the extent of the associations between CAN and MetS and/or HR remain largely unexplored. In addition, the role of MetS combined with resting HR in predicting CAN has not been well defined in the general population. In this study we hypothesize that MetS and HR associate with CAN in the population level, and the two factors have high predictive value for the outcome. Therefore, the purpose of this study is to evaluate the extent of CAN’s associations with MetS and resting HR, and to assess the predictive value of MetS combined with HR for CAN in a large sample derived from a Chinese population.

## Materials and methods

### Study population

We performed a CAN factor survey on a random sample of the Chinese population. Participants were recruited from rural and urban communities in Shanghai. Survey participants with undiagnosed CAN, aged 30–80 years, were included in this study. A total of 3,012 subjects were invited to a screening visit between 2011 and 2012. Some subjects were excluded from the study to eliminate potential confounding factors that may have influenced their CA function. Briefly, the exclusion criteria were as follows: 1) history or findings of arrhythmia, hyperthyroidism or hypothyroidism; 2) pregnancy or lactation; 3) serious chronic disease, heart failure and cancer; 4) medication of controlling resting HR such as β receptor inhibitors and/or 5) serious hepatic or renal dysfunctions. Complete baseline data were obtained for 2,092 (69.46%) of the participants. Written consent was obtained from all patients before the study. This study was approved by the Ethics Committee of the Huashan Hospital, Shanghai, China.

### Measurement

The subjects were interviewed for the documentation of medical histories and medication, history of smoking habits, laboratory assessment of cardiovascular disease risk factors and standardized examination for heart rate variability (HRV). All study subjects underwent a complete clinical baseline characteristics evaluation after an eight-hour fast, which included: 1) history and physical examination; 2) heart rate and blood pressure; 3) fasting serum glucose and insulin; 4) oral glucose tolerance test (OGTT) and 5) fasting plasma lipids. Body mass index (BMI) was calculated with weight in kilograms divided by the square of height in meters. Physicians were trained in the measurement of blood pressure (BP) and in the questionnaire before the survey according to a standard protocol [16]. Fasting plasma glucose (FPG) was quantified by the glucose oxidase procedure; HbA1c was measured by ion-exchange high-performance liquid chromatography (HPLC; Bio-Rad, Hercules, CA, USA). The homeostasis model assessment insulin resistance estimate (HOMA-IR) was calculated as serum glucose (mmol/L) multiplied by plasma insulin (U/mL) and divided by 22.5. Serum total cholesterol (TC), high-density lipoprotein (HDL) cholesterol, triglyceride (TG) levels, serum creatinine (SCr), and uric acid (UA) were measured by an enzymatic method with a chemical analyzer (Hitachi 7600–020, Tokyo, Japan). Low-density lipoprotein (LDL) cholesterol levels were calculated using the Friedewald formula. The day-to-day and inter-assay coefficients of variation at the central laboratory in our hospital for all analyses were between 1% and 3%.

Short-term HRV has good reproducibility and is more practical in its application. In our large-scale population-based study, this test was used to evaluate CA function. HRV were measured non-invasively by power spectral analysis. Before CA function assessment, participants must avoid alcohol, smoking and coffee for 24 hours so as not to influence their resting status. Subjects were studied while awake in the supine position after 20 minutes of rest. Testing times were from 8:00 to 11:00 a.m. A type-I FDP-1 HRV non-invasive detecting system was used with software version 2.0 (Department of Biomedical Engineering of the Fudan University, Shanghai, China). Electrocardiography, respiratory signals, and beat-to-beat blood pressure were continually and simultaneously recorded for 15 minutes through an electrosphygmograph transducer (HMX-3C placed on the radial artery of the dominant arm) and an instrument respiration sensor. Short-term HRV analysis was performed for all subjects using a computer-aided examination and evaluation system for spectral analysis to investigate changes in autonomic regulation.

### Definition

HT was defined as BP ≥ 140/90 mmHg, or a history of hypertension medication. BMI was classified based on the Chinese criteria: normal as BMI < 24.0 kg/m^2^; overweight as 24.0 kg/m^2^ ≤ BMI < 28.0 kg/m^2^; and obese as BMI ≥ 28.0 kg/m^2^. High FPG was defined as FPG ≥ 5.6 mmol/L. Center obesity was defined using ethnicity-specific values for waist circumference (WC) of ≥ 90 cm in men and ≥ 80 cm in women [17]. TG was defined as TG ≥ 1.7 mmol/L. HDL was defined as HDL < 0.9 mmol/L in men and HDL < 1.0 mmol/L in women. DM was defined by OGTT and either HbAlc ≥ 6.5% or the use of insulin or hypoglycemic medications. MetS was diagnosed according to the updated National Cholesterol Education Program/Adult Treatment Panel III criteria (WHO Western Pacific Region obesity criteria) in individuals meeting three or more of the following [17]: 1) central obesity; 2) TG levels, > 150 mg/dl (1.7 mmol/l) or specific treatment for this lipid abnormality; 3) HDL cholesterol, < 40 mg/dl (1.03 mmol/l) in men and < 50 mg/dl (1.29 mmol/l) in women or specific treatment for this lipid abnormality; 4) raised BP, systolic BP > 130 mm Hg or diastolic BP > 85 mm Hg or treatment for previously diagnosed HT; and 5) raised FPG level, > 100 mg/dl (> 5.6 mmol/l) or previously diagnosed type 2 DM. CAN was diagnosed based on at least two abnormal cardiovascular autonomic reflex test results from HRV tests [3].

### Statistical analysis

The Kolmogorov-Smirnov test was used to determine whether continuous variables followed a normal distribution. Variables that were not normally distributed were log-transformed to approximate normal distribution for analysis. The results are expressed as the mean ± SD or median, unless otherwise stated. The characteristics of the subjects according to CAN groups were assessed using one-way analysis of variance (ANOVA) for continuous variables and the *χ*^2^ test for categorical variables. Considering categorized variables were easier to be accepted and understood in clinical practice, in our analysis the main independent variables for CAN were MetS (categorized into two groups: code 0 for non-MetS and code 1 for MetS), resting HR (categorized into four groups: code 0 for < 65; 1 for 65–75; 2 for 75–85; and 3 for > 85 bpm) and MetS-HR (categorized into eight groups: code 0 for HR < 65 bpm and non-MetS; 1 for HR < 65 bpm and MetS; 2 for HR 65–75 bpm and non-MetS; 3 for HR 65–75 bpm and MetS; 4 for HR 75–85 bpm and non-MetS; 5 for HR 75–85 bpm and MetS; 6 for HR > 85 bpm and non-MetS; 7 for HR > 85 bpm and MetS). Univariate logistic regression was performed to determine the variables associated with CAN and to estimate confounding factors possibly disturbing the relationship between CAN and MetS or HR. Multivariate logistic linear regression (MLR) was carried out to determine the independent contributions of variables to CAN (using subjects without CAN as a reference group). Potential confounding variables were controlled in the regression model. The predictive performance of the MetS-HR was evaluated using the area under the curve (AUC) in a receiver operating characteristics (ROC) curve. Odds ratios (ORs) with 95% confidence intervals (CIs) were calculated for the relative risk of MetS, HR or MetS-HR with CAN. The results were analyzed using the Statistical Package for Social Sciences for Windows, version 16.0 (SPSS, Chicago, IL, USA). The tests were two-sided, and a p value of < 0.05 was considered significant.

## Results

The baseline clinical characteristics of the 2,902 subjects were grouped according to CAN (Table 1). The entire sample included 705 men and 1,387 women (mean age 60.42 ± 8.68 years; Table 1). A total of 387 (18.51%) subjects had CAN. There was no significant difference between subjects with CAN and those without CAN (male 32.96% vs. 36.95%, p = 0.134). The mean FPG, TC and TG levels in the total sample were 5.53 mmol/L, 5.32 mmol/L and 1.71 mmol/L, respectively. The HRV indices decreased with age (data not shown). The HR of subjects with CAN was very significantly higher than that of subjects without CAN (79.70 bpm vs. 70.77 bpm, p < 0.001). Most HRV indices were lower in subjects with CAN compared with those without CAN (p < 0.01 for all). The prevalence of HT, DM and MetS in the entire sample were 46.65%, 21.33%, and 39.82%, respectively.

**Table 1:** Clinical characteristics of subjects

**Variable**	**Entire sample**	**Subjects without CAN**	**Subjects with CAN**	***P *** value*
Demographic information				
N	2096	1705	387	
Age (years)	60.42 ± 8.68	59.85 ± 8.64	62.94 ± 8.43	<0.001
Gender (male,%)	705(33.7%)	562(32.96%)	143(36.95%)	0.134
BMI (kg/m^2^)	24.21 ± 3.37	24.07 ± 3.28	24.84 ± 3.7	<0.001
WC (cm)	85.07 ± 9.77	84.47 ± 9.62	87.72 ± 9.99	<0.001
SBP (mmHg)	127.62 ± 18.77	126.39 ± 18.22	133.05 ± 20.19	<0.001
DBP (mmHg)	79.83 ± 9.74	79.5 ± 9.65	81.31 ± 10.01	0.001
Laboratory measurement				
FPG (mmol/L)	5.53 ± 1.82	5.4 ± 1.58	6.12 ± 2.54	<0.001
PBG (mmol/L)	7.67 ± 3.63	7.36 ± 3.3	9.07 ± 4.6	<0.001
HbAlc (%)	6 ± 1.08	5.89 ± 0.92	6.47 ± 1.54	<0.001
FINS uml	7.19 ± 11.86	6.74 ± 8.03	9.18 ± 21.71	<0.001
IR (mmol/L)	1.81 ± 3.31	1.64 ± 2.13	2.54 ± 6.22	<0.001
TC (mmol/L)	5.32 ± 1	5.31 ± 0.98	5.39 ± 1.05	0.142
TG (mmol/L)	1.71 ± 0.98	1.67 ± 0.93	1.9 ± 1.17	<0.001
HDL (mmol/L)	1.36 ± 0.32	1.36 ± 0.33	1.34 ± 0.32	0.203
LDL (mmol/L)	3.19 ± 0.77	3.18 ± 0.76	3.23 ± 0.81	0.229
SCr (μmol/L)	77.81 ± 26.11	77.65 ± 26.96	78.51 ± 21.98	0.561
UA (μmol/L)	281.21 ± 84.01	280.13 ± 83.47	285.99 ± 86.26	0.216
HRV indices				
HR (bpm)	72.42 ± 10.13	70.77 ± 9.08	79.7 ± 11.26	<0.001
TP (ms^2^)	873.95 ± 702.47	1000.63 ± 693.2	315.87 ± 410.75	<0.001
LF (ms^2^)	190.98 ± 207.88	224.34 ± 215.08	43.97 ± 57.29	<0.001
HF (ms^2^)	183.05 ± 219.43	215.11 ± 229.61	41.82 ± 59.63	<0.001
LF/HF	1.7 ± 1.98	1.55 ± 1.48	2.37 ± 3.32	<0.001
Medical history				
Smoking (yes,%)	306(14.63%)	244(14.31%)	62(16.02%)	0.39
MetS (yes,%)	833(39.82%)	629(36.89%)	204(52.71%)	<0.001
DM (yes,%)	446(21.33%)	307(18.02%)	139(35.92%)	<0.001
HT (yes,%)	976(46.65%)	735(43.11%)	241(62.27%)	<0.001

Note: *present the difference between subjects with and without cardiovascular autonomic neuropathy (CAN). BMI- Body mass index, WC-waist circumference, SBP- systolic blood pressure, DBP- diastolic blood pressure, FPG- fasting plasma glucose, PBG- plasma blood glucose, FINS- fasting blood insulin, IR-insulin resistance, TC- serum total cholesterol, TG- triglyceride, UA- uric acid, HDL- high-density lipoprotein cholesterol, LDL- low density lipoprotein cholesterol, SCr- serum creatinine, HR-heart rate, TP-total power of variance, LF-low frequency, HF-high frequency, MetS- metabolic syndrome, HT- Hypertension, DM- Diabetes.

### CAN prevalence

**Figure 1:**
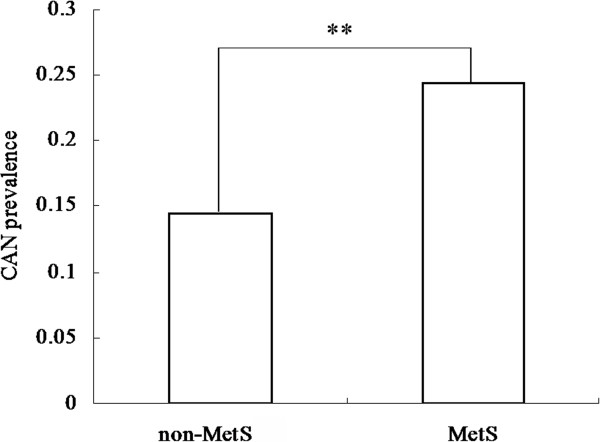
**Cardiovascular autonomic neuropathy (CAN) prevalence according to metabolic syndrome (MetS).** The CAN prevalence was 14.54% and 24.49% in respective groups according to MetS. P value for trend was less then 0.001.

**Figure 2:**
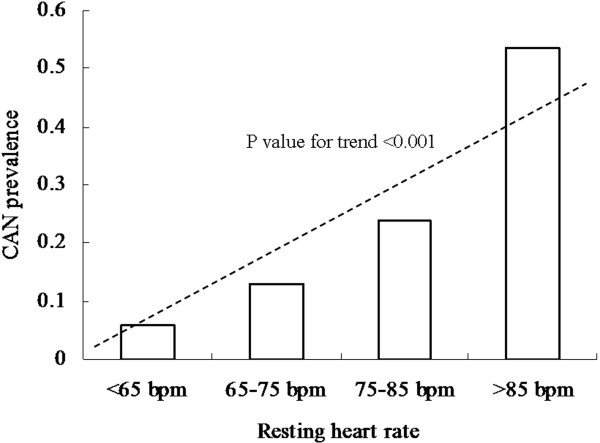
**Cardiovascular autonomic neuropathy (CAN) prevalence according to resting heart rate (HR).** The CAN prevalence was 5.92%, 12.93%, 23.94% and 53.67% in respective groups according to HR. P value for trend was less then 0.001.

**Figure 3:**
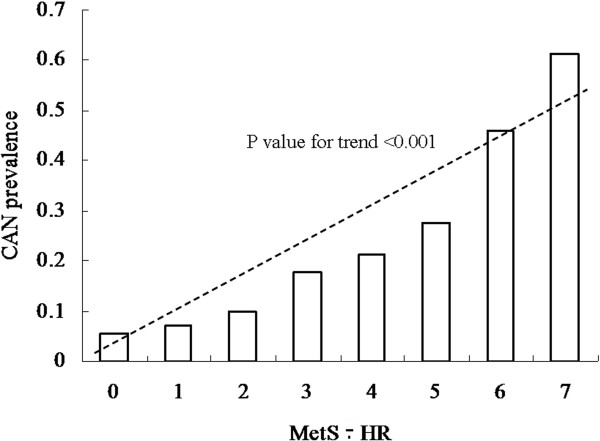
**Cardiovascular autonomic neuropathy (CAN) prevalence according to the variable of metabolic syndrome (MetS) combined with resting heart rate (MetS-HR).** The CAN prevalence was 5.32%, 7.23%, 9.8%, 17.81%, 21.15%, 27.38%, 45.87% and 61.46% in respective groups according to MetS-HR. P value for trend was less then 0.001.

The CAN prevalence was 18.50% in the total sample and 14.54% and 24.49% in the non-MetS and MetS groups, respectively. The CAN prevalence significantly increased in patients with the MetS (p < 0.001, Figure 1). CAN prevalence was 5.92%, 12.93%, 23.94% and 53.67% in the respective groups according to HR (Figure 2). There was an increased CAN prevalence trend in groups with increased HR (p for trend < 0.001). In addition, CAN prevalence significantly differed among the groups according to the categorical variable of MetS-HR (MetS-HR: 0 = 5.32%; 1 = 7.23%; 2 = 9.8%; 3 = 17.81%; 4 = 21.15%; 5 = 27.38%; 6 = 45.87%; 7 = 61.46%; Figure 3). As the MetS-HR score increased, the CAN prevalence also increased (p for trend < 0.01; Figure 3).

### Univariate analysis for CAN

To estimate the potential risk factors of CAN, univariate analysis was performed in the entire sample. These potential risk factors contained the demographic parameters, blood glucose, and insulin function parameters as well as lipid profiles and medical history factors. The results indicate that 15 potential risk factors — age, BMI, WC, SBP, DBP, FPG, PBG, HbAlc, FINS, IR, TG, HR, HT, DM and MetS — were significantly associated with CAN (p < 0.05 for all parameters; Table 2). The two variables of HR and MetS were very significantly associated with CAN in a univariate analysis model.

**Table 2:** Univariate logistic regression analysis for cardiovascular autonomic neuropathy

**Variable**	**β**	**S.E.**	**P value**	**OR**	**95% CI**
Age	0.042	0.007	<0.001	1.043	1.029–1.103
Gender	0.176	0.117	0.134	1.192	0.947–1.547
BMI	0.066	0.016	<0.001	1.068	1.034–1.046
WC	0.034	0.006	<0.001	1.034	1.023–1.024
SBP	0.018	0.003	<0.001	1.018	1.012–1.030
DBP	0.019	0.006	0.001	1.019	1.007–1.261
FPG	0.178	0.027	<0.001	1.195	1.133–1.149
PBG	0.111	0.014	<0.001	1.117	1.087–1.722
HbAlc	0.392	0.077	<0.001	1.48	1.271–1.026
FINS	0.014	0.006	0.015	1.014	1.003–1.159
IR	0.091	0.029	0.001	1.095	1.036–1.212
TC	0.082	0.056	0.142	1.086	0.973–1.369
TG	0.213	0.051	<0.001	1.238	1.119–1.129
HDL	-0.225	0.177	0.203	0.798	0.564–1.259
LDL	0.088	0.073	0.229	1.092	0.946–1.005
UA	0.001	0.001	0.216	1.001	0.999–1.108
Smoking	0.133	0.155	0.39	1.142	0.843–2.733
HT	0.779	0.116	<0.001	2.178	1.736–3.248
DM	0.936	0.123	<0.001	2.550	2.003–3.156
HR	0.952	0.068	<0.001	2.590	2.267–2.565
MetS	0.646	0.114	<0.001	1.907	1.527–1.307
MetS-HR	0.469	0.033	<0.001	1.598	1.499–1.500

Note: Cardiovascular autonomic neuropathy (CAN). BMI- Body mass index, WC-waist circumference, SBP- systolic blood pressure, DBP- diastolic blood pressure, FPG- fasting plasma glucose, PBG- plasma blood glucose, FINS- fasting blood insulin, IR-insulin resistance, TC- serum total cholesterol, TG- triglyceride, UA- uric acid, HDL- high-density lipoprotein cholesterol, LDL- low density lipoprotein cholesterol, SCr- serum creatinine, HR-heart rate, TP-total power of variance, LF-low frequency, HF-high frequency, MetS- metabolic syndrome, HT- Hypertension, DM- Diabetes.

### Multivariate analysis for CAN

To estimate the association of MetS and HR or the categorical variable of MetS-HR with CAN, univariate logistic regression models were developed to include age, gender, BMI, WC, SBP, DBP, FPG, TG, HDL, other lipid profiles, SCr, UA and medical history (Table 2). MLR analysis was carried out to determine the extent to which CAN was associated with MetS and HR. MetS and HR remained very significantly associated with CAN after adjustments for age, gender, smoking, LDL, SCr and UA (p < 0.001 for MetS and HR, respectively). In subjects with HR ranged from 65 to 75 bpm, the OR of CAN was 2.592 (95% CI, 2.260-2.973; p < 0.001; Table 3) compared to subjects with HR < 65 bpm. And in subjects with MetS, the OR of CAN was 1.641 (95% CI, 1.282-2.099; p < 0.001; Table 3) compared to subjects without MetS. In addition, a very significant association between MetS-HR and CAN was found by using MLR adjustment for potential confounds including age, gender, smoking, LDL, SCr and UA (p < 0.001). In subjects with MetS-HR of 1, the OR of CAN was 1.612 (95% CI, 1.510-1.722; p < 0.001; Table 3) compared to subjects with MetS-HR of 0.

**Table 3:** Multivariate logistic regression analysis for cardiovascular autonomic neuropathy

**Model**	**Variable**	**β**	**S.E.**	**P value**	**OR**	**95% CI**
Model 1	HR	0.952	0.07	<0.001	2.592	2.260–2.973
	MetS	0.495	0.126	<0.001	1.641	1.282–2.099
Model 2	MetS-HR	0.477	0.033	<0.001	1.612	1.510–1.722

Note: Model 1 and Model 2 adjusted for age, gender, smoking, SCr, UA, LDL; SCr- serum creatinine, UA- uric acid, LDL- low density lipoprotein cholesterol.

### Predictive value analysis for CAN

**Figure 4:**
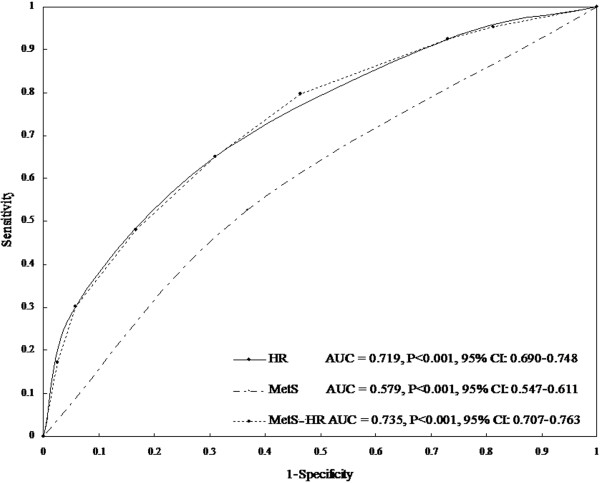
**Receiver operating characteristic curves showed the performance of resting heart rate (HR), metabolic syndrome (MetS) and categorical variable of MetS + HR in predicting cardiovascular autonomic neuropathy (CAN) prevalence in this dataset.** The 95% confidence interval (CI) is given in parentheses. AUC represents area under the curve. HR: AUC = 0.719 (95% CI : 0.690-0.748), P < 0.001; MetS: AUC = 0.579 (95% CI: 0.547-0.611), P < 0.001; MetS-HR: AUC = 0.735 (95% CI: 0.707-0.763), P < 0.001.

To evaluate the predictive performance of MetS, HR and MetS-HR for CAN, the AUC in an ROC curve was calculated. For the MetS variable, the AUC was 0.579 (95% CI: 0.547-0.611, p < 0.001), indicating that MetS moderately predicted CAN (Figure 4). Whereas for both the HR and MetS-HR variables, the AUC was 0.719 (95% CI: 0.690-0.748, p < 0.001) and 0.735 (95% CI: 0.707-0.763, p < 0.001), respectively, suggesting that HR and MetS-HR strongly predicted CAN (Figure 4). A cut-off point for MetS-HR was set to 4 of 7, and the sensitivity and specificity of CAN were 65.10% and 68.90% (Youden index = 0.340), respectively. The sensitivity and specificity of CAN were 79.80% and 53.50% (Youden index = 0.333), respectively, when the cut-off point was set to 3 of 7.

## Discussion

A large-scale population-based cross-sectional study was conducted to evaluate the extent to which MetS and resting HR are associated with CAN among 2,092 participants in the Chinese population. Importantly, we first performed a predictive value analysis for CAN by using resting HR combined with MetS. This sample was a good representation of the Chinese population, and the findings may work similarly well outside the areas studied in China. It is crucial for us to understand the predictive value of MetS and resting HR on CAN in the general population. This is partly because the prevalence of MetS has increased rapidly in China, and may also contribute to CAN progression. Clinicians can expect to treat more MetS patients having CAN progression. Moreover, a better understanding the predictive value of the two factors will help clinicians in preventing and treating CAN.

The main finding of the present study is that MetS and resting HR are strongly and independently associated with CAN in the general population. In this study, CAN prevalence analysis indicated that its prevalence increased with increased resting HR (p for trend < 0.001, Figure 2). CAN prevalence was 53.67% in subjects with resting HR > 85 bpm, while its prevalence was 5.92% in subjects with resting HR < 65 bpm. A higher CAN prevalence was found in subjects with MetS as compared to subjects without MetS (p < 0.001, Figure 1). Univariate analysis showed that MetS and resting HR was very significantly associated with CAN (p < 0.001 for both, Table 2), respectively. Moreover, after adjustment for potential confounds, MLR analysis demonstrated that MetS and resting HR very significantly and independently remain associated with CAN (β = 0.495 for MetS and β = 0.952 for HR, p < 0.001 for both, Table 3). These results provided strong evidence to support the hypothesis of a good association of CAN with MetS and resting HR.

Our previous study [15] and other previous studies reported that MetS is significantly associated with CA function indices. Garruti et al. [4] conducted a hospital-based study in 180 diabetic patients to indicate that MetS was more strongly associated with CA dysfunction than isolated DM. Another hospital-based study reported that disturbed HRV indices were present in patients with MetS before the development of DM [13]. The two studies were hospital-based studies and conducted in a small specific sample, which had selection bias and less power to detect associations. However, our study was based on a large-scale Chinese population to have power enough to detect associations, which keep this study from hospital selection bias. Chang et al. [14] conducted a large-scale study to explore the relationship of metabolic factors with CA function to show that CA function altered in pre-disease subjects with one or more metabolic abnormalities. This study was conducted in a specific group, mainly subjects with diabetes and pre-diabetes. Our study extended the specific sample to general population, which had more significant in clinical practice. As mentioned above, these studies showed evidence that MetS is strongly associated with CAN in different groups, indicating that our results were consistent with these studies. Studies have previously shown that resting HR is a critical factor and strong predictor of CAN in diabetic patients [11,12]. Generally, increased resting HR was considered one of early indicators of CAN. The clustering of cardiovascular risk factors in MetS indicates that the multiple complex metabolic reactions involved in glycotoxicity, lipotoxicity, altered insulin signaling, increased cytokine activity and interstitial deposition of triacylglycerol may directly or indirectly affect CA function [18-23]. Additionally, these metabolic risk factors lead to reduced energy availability and have an added adverse effect on endothelial function [24].

Another interesting finding was that resting HR alone and combined with MetS had a high value in predicting CAN in the general population. As mentioned above, resting HR was very significantly and independently associated with CAN. The AUC was calculated to show that this factor strongly predicts CAN (AUC = 0.719, 95% CI: 0.690-0.748). For the analysis of the predictive value of MetS alone on CAN, although association analysis showed that MetS was very significantly and independently associated with CAN, the AUC was calculated to indicate that MetS moderately predicts CAN (AUC = 0.579, 95% CI: 0.547-0.611). However, we used a categorical variable of MetS-HR, which combined information between resting HR and MetS, to signify a high value in predicting CAN in the general population (AUC = 0.735, 95% CI: 0.707-0.763). The sensitivity and specificity of CAN were 65.10% and 68.90% when the optimal cut-off point of MetS-HR was set to 4 of 7. When the cut-off point of MetS-HR was 3, a higher sensitivity of CAN but a lower specificity was reported. In particular, the CAN prevalence was 61.46% in subjects with resting HR > 85 bpm and MetS (MetS-HR = 7), while its prevalence decreased to 5.32% in subjects with resting HR < 65 bpm without MetS (MetS-HR = 1). These results provide evidence that resting HR and MetS-HR have a high value in predicting CAN in the general Chinese population. To our knowledge, this is the first study to have reported resting HR combined with MetS having such a high predictive value for CAN in a Chinese population. These results provided evidence to support the hypothesis that MetS combined resting HR has a high predictive value for CAN. This finding is of importance to the clinical practice of preventing and treating CAN in the general population.

The categorical variable MetS-HR combines information on MetS and resting HR, but it cannot obtain a sensitivity of 100%. A false negative is mainly attributed to the fact that other risk factors contribute to the outcome. In this study, some of the patients with CAN were not obese, or had a normal resting HR due to both impaired sympathetic and parasympathetic nervous systems. Little is known about the CAN prevalence in subjects with a normal resting HR in the population. In addition, false-negative individuals had a lower resting HR, indicating that those people had long-term CAN. The exact mechanism underlying the association between CAN and MetS or resting HR has not been fully elucidated. In the present study, we did not determine the mechanism by which TG modifies metabolic factors and induces DHF.

Several limitations of this study deserve comment. First, the study design was cross-sectional, and thus the temporal sequence between risk factors and outcome was questionable. In addition, this prediction model was both derived and tested within the same cohort. So this model should be verified in external dataset. Finally, it is important to mention that our study was performed on Chinese individuals, and our findings may not be relevant to people of other ethnicities.

In conclusion, a higher CAN prevalence was frequently found in subjects with increased resting HR and/or MetS. There was a tendency toward increased CAN prevalence with increased resting HR. Our findings signify that resting HR and MetS are independently associated with CAN, and resting HR alone and combined with MetS both have a high predictive value in predicting CAN in the general population. These observations provide evidence that provide novel insights into biological functions in the future.

## Abbreviations

BMI: Body mass index; BSA: Body surface area; CI: Confidence intervals; CAN: Cardiovascular autonomic neuropathy; SCr: Serum Creatinine; DBP: Diastolic blood pressure; DM: Diabetes; FPG: Fasting plasma glucose; HbAlc: Glycosylated hemoglobin; HDL: High-density lipoprotein cholesterol; HOMA-IR: Homeostasis model assessment insulin resistance estimate; HRV: Heart rate variability; HT: Hypertension; IDF: International Diabetes Federation; LDL: Low-density lipoprotein cholesterol; MetS: Metabolic syndrome; MLR: Multivariable logistic linear regression; OGTT: Oral glucose tolerance test; OR: Odds ratios; PBG: Postprandial blood glucose; HT: Hypertension; SBP: Systolic blood pressure; TC: Serum total cholesterol; TG: Triglyceride; WC: Waist circumference; UA: Uric acid.

## Competing interests

The authors declare that they have no conflicts of interests.

## Authors’ contribution

YL, ZL and FZ carried out the molecular genetic studies, participated in the sequence alignment and drafted the manuscript. YL, ZHT and LZ participated in the design of the study and performed the statistical analysis. YL and LZ conceived of the study, and participated in its design and coordination and helped to draft the manuscript. All authors read and approved the final manuscript.
